# Why Targeting Tumor Acidity Fails: Translational Barriers and Emerging Solutions

**DOI:** 10.3390/ijms27104623

**Published:** 2026-05-21

**Authors:** Kyung-Hee Kim, Byong Chul Yoo

**Affiliations:** 1 Department of Applied Chemistry, School of Science and Technology, Kookmin University, Seoul 02707, Republic of Korea; kyungheekim@kookmin.ac.kr; 2 Antibody Research Institute, Kookmin University, Seoul 02707, Republic of Korea; 3 Diagnostic Research Team, InnoBation Bio R&D Center, Seoul 03929, Republic of Korea

**Keywords:** tumor acidity, tumor microenvironment, lactate metabolism, metabolic plasticity, immunosuppression, translational barriers

## Abstract

Tumor acidity is a hallmark of the tumor microenvironment (TME) and has been widely regarded as a promising therapeutic target due to its ubiquity, functional relevance, and apparent selectivity for malignant tissues. Extensive preclinical studies have demonstrated that targeting tumor acidity—through inhibition of lactate production, blockade of proton transport, systemic buffering, and pH-responsive drug delivery—can suppress tumor growth, reduce metastasis, and enhance antitumor immunity. However, despite strong mechanistic rationale and consistent preclinical efficacy, these strategies have failed to achieve meaningful and durable clinical success. In this review, we examine the underlying reasons for this translational discrepancy. We highlight key mechanistic and systemic barriers, including spatial heterogeneity of tumor pH, temporal dynamics and adaptive evolution, metabolic plasticity, redundancy of pH-regulating systems, systemic physiological constraints, and drug delivery limitations in hypoxic and acidic regions. We further argue that tumor acidity is not a sufficient standalone driver of tumor progression but rather a feature of a complex and adaptive system shaped by metabolic and microenvironmental interactions. Finally, we discuss emerging strategies that may overcome these limitations, including combination therapies integrating metabolic targeting with immunotherapy, pH-responsive drug delivery systems, microenvironment reprogramming, and biomarker-guided patient stratification. Overall, current evidence suggests that future therapeutic approaches may benefit more from exploiting tumor acidity as a feature of the tumor microenvironment rather than attempting to directly neutralize it.

## 1. Introduction

The tumor microenvironment (TME) is increasingly recognized as a critical determinant of cancer progression, therapeutic response, and immune regulation. Among its defining features, extracellular acidification represents one of the most consistent and evolutionarily conserved hallmarks across diverse tumor types [1,2,3,4]. Unlike normal tissues, which tightly regulate extracellular pH within a narrow physiological range, solid tumors commonly exhibit a reversed pH gradient characterized by intracellular alkalinization and extracellular acidification, with interstitial pH values frequently dropping below 6.8 [5,6,7,8].

This acidic microenvironment arises primarily from metabolic reprogramming, particularly the preferential utilization of aerobic glycolysis, also known as the Warburg effect [2,9,10,11]. Enhanced glycolytic flux leads to the accumulation of lactate and protons, which are actively exported into the extracellular space via monocarboxylate transporters (MCTs), Na^+^/H^+^ exchangers, and carbonic anhydrases [8,12,13]. In addition, inadequate perfusion and hypoxia further exacerbate acidification by promoting glycolysis and limiting the clearance of acidic metabolites [14,15,16]. Importantly, stromal components—including cancer-associated fibroblasts (CAFs), immune cells, and endothelial cells—also contribute to metabolic acid production, reinforcing the notion that tumor acidity is a collective property of the TME rather than a tumor cell-intrinsic phenomenon [17,18,19].

Functionally, extracellular acidosis exerts profound effects on tumor biology. Acidic conditions promote local invasion by facilitating extracellular matrix degradation and enhancing cell motility [6,20]. Furthermore, tumor acidity has emerged as a potent regulator of immune suppression, impairing cytotoxic T lymphocyte and natural killer cell function while promoting the accumulation of regulatory T cells and myeloid-derived suppressor cells [19,21,22]. At the same time, acidosis contributes to therapeutic resistance by altering drug uptake, modifying drug stability, and inducing adaptive stress responses in cancer cells [23,24,25]. Collectively, these observations have led to the widespread view that tumor acidity is not merely a byproduct of cancer metabolism but a driver of malignant progression and a compelling therapeutic target.

Based on this strong biological rationale, numerous strategies have been developed to target tumor acidity. These include inhibition of lactate production and transport, modulation of proton extrusion systems, systemic buffering approaches, and the development of pH-responsive drug delivery systems [13,26,27,28]. In preclinical models, many of these approaches demonstrate significant antitumor efficacy, including reduced tumor growth, decreased metastatic potential, and enhanced immune activity [18,22,27]. Such findings have fueled considerable enthusiasm for translating acidity-targeting strategies into clinical applications.

However, despite decades of research and strong mechanistic support, clinical success has been limited. To date, no therapy specifically designed to neutralize or exploit tumor acidity has achieved consistent and durable efficacy across cancer types [13,25]. Clinical trials targeting metabolic pathways, proton transport systems, or tumor pH have frequently yielded modest or inconsistent outcomes, highlighting a persistent gap between preclinical promise and clinical reality. This discrepancy suggests that the current conceptual framework for targeting tumor acidity may be incomplete.

**Figure 1:**
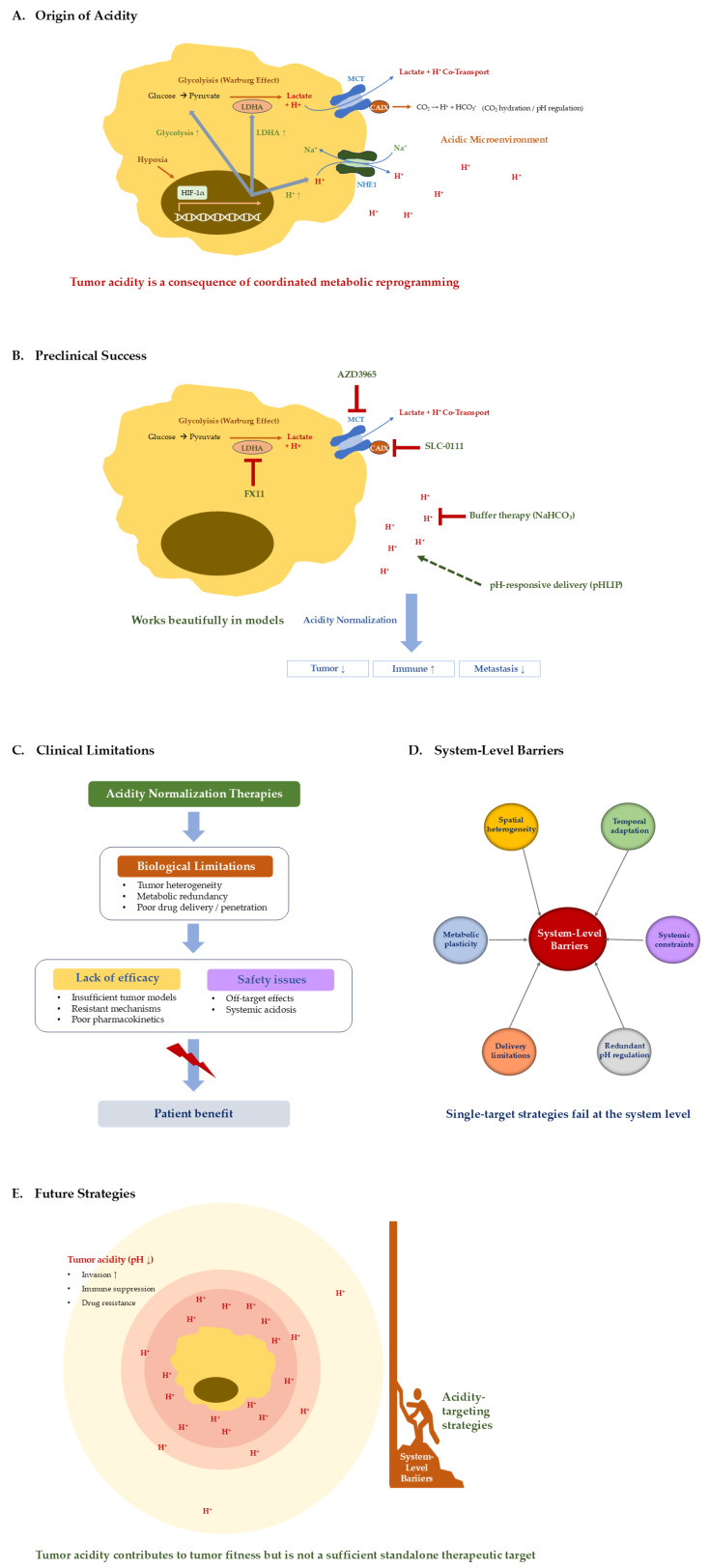
Proposed systems-level framework explaining the translational failure of tumor acidity-targeting strategies. (**A**) Schematic representation of tumor acidity within the tumor microenvironment. Tumor cells exhibit enhanced glycolytic metabolism and proton export, leading to extracellular acidification (pH ↓) and the formation of a proton gradient surrounding tumor tissue. (**B**) Functional consequences of tumor acidity. Acidic extracellular pH promotes tumor progression by enhancing invasive behavior, suppressing anti-tumor immune responses, and reducing the efficacy of anticancer drugs. (**C**) Therapeutic strategies targeting tumor acidity. Representative approaches include buffering strategies and metabolic interventions aimed at reducing proton production or accumulation. Although these approaches show promising effects in preclinical models, their clinical efficacy remains limited. (**D**) System-level barriers limiting therapeutic efficacy. Tumor acidity is sustained by a complex and interconnected network of biological processes, including metabolic plasticity, spatial and temporal heterogeneity, redundant pH-regulating mechanisms, delivery limitations, and systemic constraints. These factors collectively maintain acidity despite targeted interventions. (**E**) Conceptual framework illustrating the limitation of acidity-targeting strategies. Tumor acidity contributes to tumor fitness—promoting invasion, immune suppression, and drug resistance—but represents a downstream manifestation of system-level processes rather than an independent therapeutic node. Consequently, therapeutic strategies that directly target acidity encounter intrinsic limitations imposed by underlying system-level constraints. Effective intervention likely requires modulation of the broader biological system that generates and sustains tumor acidity. This figure summarizes the perspective discussed in this review, in which tumor acidity is viewed as a consequence of broader metabolic and microenvironmental processes rather than an isolated therapeutic target.

In this review, we examine this translational paradox and discuss why therapies targeting tumor acidity have shown limited clinical success despite strong preclinical rationale. We argue that tumor acidity is better understood as a consequence of broader metabolic and microenvironmental interactions rather than as an isolated therapeutic target. From this perspective, extracellular acidosis reflects the adaptive state of the tumor microenvironment and may therefore require integrated therapeutic approaches rather than direct pH modulation alone (Figure 1).

## 2. Biological Basis of Tumor Acidity as a Therapeutic Target

### 2.1. Universality of Tumor Acidity as a Convergent Phenotype

Tumor acidity has long been considered an attractive therapeutic target due to its ubiquity and apparent selectivity for malignant tissues. Unlike many oncogenic pathways that exhibit substantial genetic heterogeneity across tumor types, extracellular acidification represents a convergent phenotype arising from diverse oncogenic processes, including metabolic reprogramming, hypoxia, and aberrant vascularization [1,2,3,5,20,29]. This universality suggests that targeting tumor acidity could provide a broadly applicable therapeutic strategy independent of tumor genotype.

### 2.2. Reversed pH Gradient and Active Regulation of Proton Dynamics

A defining feature of solid tumors is the establishment of a reversed pH gradient, characterized by a relatively alkaline intracellular pH (pHi ~7.2–7.6) and an acidic extracellular pH (pHe ~6.3–6.9) [7,8,13,30]. This gradient is actively maintained through coordinated regulation of proton transport systems, including Na^+^/H^+^ exchangers (NHE1), monocarboxylate transporters (MCT1/4), vacuolar H^+^-ATPases (V-ATPases), and carbonic anhydrases such as CAIX [8,12,13,26,31,32]. The resulting extracellular acidification creates a biochemical environment that is fundamentally distinct from normal tissues, thereby offering a potential therapeutic window.

### 2.3. Acid-Mediated Invasion and Tissue Remodeling

Functionally, acidic pH confers multiple advantages to tumor cells, particularly in the context of invasion and metastasis. The “acid-mediated invasion hypothesis” proposes that tumor-derived acidity promotes local tissue invasion by inducing extracellular matrix degradation and selective toxicity toward surrounding normal cells [6,20,33]. Experimental studies have demonstrated that low extracellular pH enhances the activity of proteases and facilitates tumor cell dissemination [34,35].

### 2.4. Immunosuppressive Effects of Tumor Acidity

Tumor acidity also plays a critical role in shaping the immune landscape of the TME. Acidic conditions impair the effector functions of cytotoxic T lymphocytes and natural killer cells by inhibiting cytokine production, reducing proliferation, and disrupting metabolic fitness [21,22,36,37]. At the same time, acidosis promotes the accumulation and suppressive activity of regulatory T cells and myeloid-derived suppressor cells [19,38]. Lactate, a major contributor to tumor acidity, further acts as an immunomodulatory metabolite capable of directly influencing immune cell differentiation and function [19,39].

#### Tumor Acidity as an Immunometabolic Constraint

Tumor acidity exerts immunosuppressive effects not only through extracellular pH reduction itself, but also by imposing immunometabolic constraints on effector immune cells within the tumor microenvironment. Recent studies suggest that acidic lactate-rich tumor microenvironments disrupt immune-cell metabolism and reinforce T-cell dysfunction through impaired lactate handling, intracellular acid stress, and bioenergetic collapse [21,22,40].

Activated cytotoxic T lymphocytes (CTLs) require high glycolytic flux to sustain proliferation, cytokine production, and effector activity. However, glucose depletion by highly glycolytic tumor cells, combined with extracellular lactate accumulation, suppresses glycolytic fitness in T cells and reduces IFN-γ production and cytotoxic function [21,22]. Acidic conditions also impair mitochondrial fitness and metabolic flexibility in effector immune cells, thereby promoting functional exhaustion and limiting durable antitumor immunity [40].

Importantly, lactate is increasingly recognized not merely as a metabolic byproduct but as an active immunomodulatory metabolite capable of reshaping immune-cell differentiation and function [19,39]. Tumor-derived lactate promotes immunosuppressive polarization of tumor-associated macrophages, enhances regulatory T-cell stability, and impairs dendritic-cell activation [19,41]. These findings suggest that tumor acidity influences immune-cell metabolism and contributes to competition for metabolic resources within the tumor microenvironment.

### 2.5. Contribution to Therapeutic Resistance

Tumor acidity contributes to therapeutic resistance through multiple mechanisms. Weakly basic chemotherapeutic agents exhibit reduced cellular uptake in acidic environments due to ion trapping, while extracellular pH can influence drug distribution and stability [23,30]. In addition, chronic acidosis induces adaptive cellular responses—including metabolic reprogramming and stress signaling—that enhance tumor cell survival under adverse conditions [24,25,42,43].

Beyond its effects on immune suppression and invasion, extracellular acidosis also influences gene regulation and cellular metabolic programming. Acidic microenvironments have been associated with transcriptional reprogramming linked to stress adaptation, epithelial–mesenchymal transition, and therapy resistance [41,42]. Acidosis may additionally alter epigenetic regulation through metabolic effects on histone modification and chromatin-associated pathways, although these mechanisms remain incompletely understood [3,25]. Emerging evidence also suggests that acidic conditions can promote lipid metabolic remodeling, including increased fatty acid uptake and altered membrane lipid composition, potentially contributing to tumor survival under nutrient- and oxygen-limited conditions [44,45,46,47].

### 2.6. Metabolic Tractability and Therapeutic Targetability

Importantly, the metabolic origin of tumor acidity suggests that it may be therapeutically tractable. The dependence of many tumors on aerobic glycolysis results in high rates of lactate production mediated by enzymes such as lactate dehydrogenase A (LDHA) and exported via monocarboxylate transporters [2,26,48]. Inhibition of these pathways has been shown to reduce lactate accumulation, normalize extracellular pH, and impair tumor growth in preclinical models [18]. Similarly, targeting proton transport systems or buffering tumor acidity has demonstrated promising antitumor effects in animal studies [13,27].

### 2.7. Summary: A Seemingly Ideal Therapeutic Target

Collectively, tumor acidity fulfills several key criteria of an ideal therapeutic target: it is widespread across tumor types, functionally relevant to cancer progression, mechanistically linked to multiple hallmarks of cancer, and potentially targetable through diverse pharmacological strategies [13,29,49]. These features have led to the widespread assumption that tumor acidity represents a “druggable” vulnerability. However, as discussed in the following sections, this expectation has not been realized in clinical practice. These mechanisms are summarized in Table 1.

## 3. Preclinical Evidence Supporting Acidity-Targeting Strategies

### 3.1. Targeting Lactate Production and Metabolic Reprogramming

One of the most extensively explored strategies in preclinical models involves targeting lactate production through inhibition of key metabolic enzymes. Lactate dehydrogenase A (LDHA), which catalyzes the conversion of pyruvate to lactate, has been a primary focus due to its central role in maintaining high glycolytic flux in cancer cells [2]. Pharmacological or genetic inhibition of LDHA reduces lactate production, induces oxidative stress, and suppresses tumor growth in multiple preclinical systems [57,58].

In addition to LDHA, modulation of upstream metabolic pathways—such as pyruvate dehydrogenase activation using dichloroacetate (DCA)—has demonstrated the ability to shift cancer cell metabolism toward oxidative phosphorylation, thereby reducing lactate accumulation [59]. These findings have contributed to the concept that tumor acidity can be attenuated by reprogramming cancer metabolism.

### 3.2. Inhibition of Lactate Transport and Metabolic Symbiosis

Beyond lactate production, targeting lactate transport has emerged as a powerful strategy in preclinical models. Monocarboxylate transporters (MCTs), particularly MCT1 and MCT4, facilitate the export and exchange of lactate between tumor cells and the surrounding microenvironment [12,26]. Inhibition of MCT1 disrupts the metabolic coupling between hypoxic and oxygenated tumor cells, a phenomenon described as metabolic symbiosis [17,18].

Preclinical studies have shown that blocking lactate transport leads to intracellular acidification, metabolic stress, and tumor growth inhibition [18,50,60,61,62,63]. These findings suggest that interfering with lactate flux may be an effective way to disrupt tumor metabolic homeostasis and reduce extracellular acidification.

### 3.3. Systemic Buffering and Neutralization of Tumor Acidity

Another widely studied approach involves the use of systemic buffers to neutralize tumor acidity. Preclinical studies have demonstrated that oral administration of bicarbonate can increase tumor pH, reduce local invasion, and inhibit spontaneous metastasis in mouse models [27,64]. Buffer therapy has also been shown to enhance the efficacy of weakly basic chemotherapeutic agents by improving drug uptake in acidic tumor regions [23,65].

These results have been interpreted as evidence that tumor acidity is not only a marker of malignancy but also a functional driver of metastasis that can be therapeutically reversed.

### 3.4. Targeting Proton Transport and pH Regulation Systems

Tumor cells actively maintain intracellular pH homeostasis through a network of proton transporters and enzymes. Preclinical studies targeting these systems—including Na^+^/H^+^ exchangers (NHE1), vacuolar H^+^-ATPases (V-ATPases), and carbonic anhydrases such as CAIX—have demonstrated significant antitumor effects [13,31,51,66].

Inhibition of these transporters disrupts proton extrusion, leading to intracellular acidification and reduced tumor cell viability [7,8]. Notably, CAIX has been extensively studied as a hypoxia-inducible enzyme that contributes to extracellular acidification, and its inhibition has shown promising results in preclinical cancer models [31,52,67].

### 3.5. pH-Responsive Drug Delivery and Tumor-Selective Targeting

Tumor acidity has also been exploited as a trigger for selective drug delivery. A variety of pH-responsive nanoparticles, polymers, and prodrugs have been developed to release therapeutic agents specifically in acidic environments [28,68,69,70]. These systems take advantage of the pH differential between tumor and normal tissues to enhance tumor specificity and reduce systemic toxicity.

Preclinical studies have demonstrated improved drug accumulation, enhanced therapeutic efficacy, and reduced off-target effects using pH-sensitive delivery platforms [68,69,71]. These findings further reinforce the potential of tumor acidity as a clinically exploitable feature.

### 3.6. Modulation of the Immune Microenvironment

Recent studies have highlighted the role of tumor acidity in immune suppression and the potential for metabolic interventions to restore immune function. Inhibition of lactate production or transport has been shown to enhance T cell proliferation, increase cytokine production, and improve the efficacy of immune checkpoint blockade in preclinical models [19,21,22].

These findings have led to the emergence of combination strategies in which targeting tumor metabolism is used to sensitize tumors to immunotherapy, further expanding the therapeutic implications of tumor acidity.

### 3.7. Summary: Consistent Preclinical Efficacy Across Diverse Strategies

Taken together, preclinical studies across multiple independent approaches—including metabolic inhibition, transport blockade, buffering, proton pump targeting, and pH-responsive drug delivery—consistently demonstrate antitumor efficacy. These strategies not only reduce tumor growth but also inhibit metastasis, enhance immune responses, and improve drug delivery.

This remarkable consistency has contributed to a strong and widely accepted narrative: tumor acidity is a critical driver of cancer progression and a therapeutically actionable vulnerability. However, the uniform success of these strategies in preclinical models stands in stark contrast to their limited clinical translation. This discrepancy suggests that preclinical systems may overestimate the therapeutic impact of targeting tumor acidity, raising the possibility that these models capture only a subset of the complexity present in human tumors. These preclinical strategies are summarized in Table 2.

## 4. Clinical Translation: Limited Efficacy Despite Strong Rationale

### 4.1. Metabolic Targeting: Biochemical Activity Without Clinical Efficacy

Targeting tumor metabolism, particularly lactate production, has been one of the most actively pursued strategies in clinical settings. Agents such as dichloroacetate (DCA), which promotes oxidative phosphorylation by activating pyruvate dehydrogenase, have demonstrated measurable metabolic effects in experimental and translational settings, including altered metabolic flux [59,73]. Similarly, inhibitors of lactate dehydrogenase A (LDHA) have shown promising preclinical activity and have advanced into early-phase clinical investigation.

However, despite clear pharmacodynamic effects, these approaches have not translated into consistent clinical benefit. Tumor responses have been modest, heterogeneous, and often transient. One explanation is that cancer cells exhibit substantial metabolic plasticity, allowing them to compensate for inhibition of glycolysis by increasing oxidative phosphorylation or utilizing alternative substrates [2,3,72]. As a result, targeting a single metabolic pathway may be insufficient to disrupt the overall metabolic network that sustains tumor growth.

### 4.2. Inhibition of Lactate Transport: Context-Dependent Responses

Monocarboxylate transporters, particularly MCT1, have emerged as clinically relevant targets, with inhibitors such as AZD3965 entering early-phase trials [61,78]. These agents are designed to block lactate export and disrupt metabolic symbiosis within the tumor microenvironment.

While early clinical studies have demonstrated target engagement and alterations in tumor metabolism, antitumor efficacy has been limited and highly context-dependent. In particular, the expression of alternative transporters such as MCT4 can compensate for MCT1 inhibition, reducing therapeutic effectiveness [26,50,79,80,81]. Moreover, the reliance on lactate as a metabolic substrate varies across tumor types and even within different regions of the same tumor, further complicating therapeutic targeting [80,82].

### 4.3. Buffer Therapy: Systemic Constraints and Limited Translational Feasibility

Buffering strategies aimed at neutralizing tumor acidity have shown strong antitumor effects in preclinical models but have faced significant challenges in clinical translation. The administration of systemic buffers, such as sodium bicarbonate, is limited by physiological constraints on systemic pH regulation, making it difficult to achieve sustained and tumor-specific modulation of extracellular acidity [27,65].

In clinical settings, tolerability issues, dosing limitations, and the lack of tumor selectivity have restricted the applicability of buffering approaches. Furthermore, systemic buffering may have unintended effects on normal tissues, highlighting the difficulty of targeting a parameter—pH—that is tightly regulated at the organismal level [64,83].

### 4.4. Targeting Proton Transport Systems: Redundancy and Limited Specificity

Inhibitors of proton transport systems, including carbonic anhydrase IX (CAIX), vacuolar H^+^-ATPases, and Na^+^/H^+^ exchangers, have been explored as potential therapeutic agents [31,51,52,84,85]. These targets are appealing due to their role in maintaining the reversed pH gradient characteristic of tumor cells.

However, clinical outcomes have been inconsistent, and several challenges have emerged. First, the redundancy of pH-regulatory mechanisms allows tumor cells to compensate for inhibition of a single transporter [13,42]. Second, many of these targets are not exclusively expressed in tumor tissues, raising concerns about off-target effects and toxicity [86]. Third, drug delivery to hypoxic and poorly perfused tumor regions—where acidity is most pronounced—remains a significant barrier [53,54,55].

### 4.5. Hypoxia-Targeted Therapies: Variable Efficacy and Patient Selection Challenges

Given the close relationship between hypoxia and tumor acidity, hypoxia-targeted therapies have been considered a complementary approach. Hypoxia-activated prodrugs and inhibitors of hypoxia-inducible factors (HIFs) have demonstrated activity in certain tumor types but have produced variable results in clinical trials [87,88,89,90,91].

One of the primary challenges is the spatial and temporal heterogeneity of hypoxia within tumors. Regions of hypoxia—and thus acidity—are not uniformly distributed, making it difficult to achieve consistent therapeutic effects [92,93,94]. Additionally, the lack of reliable biomarkers for patient selection further limits the clinical impact of these approaches [91].

### 4.6. Pharmacodynamic Success Does Not Equal Clinical Benefit

A recurring theme across clinical studies is the disconnect between pharmacodynamic activity and clinical efficacy. Many acidity-targeting interventions successfully modulate biochemical parameters—such as lactate levels, pH gradients, or metabolic flux—yet fail to produce meaningful tumor regression or survival benefits [13,25,86].

This observation suggests that tumor acidity, while biologically important, may not function as a dominant driver of tumor growth in the clinical setting. Alternatively, it may indicate that the current strategies used to target acidity do not adequately capture the complexity of its role within the tumor microenvironment [29,95].

#### Clinical and Translational Determinants of Therapeutic Failure

Several factors may explain why acidity-targeting strategies that appear effective in preclinical systems fail to produce meaningful clinical benefit. One major limitation is the absence of reliable patient selection strategies. Tumors differ substantially in their dependence on glycolysis, lactate metabolism, hypoxia, and proton transport activity, yet most clinical studies have not incorporated biomarker-guided stratification [3,87,96]. As a result, therapies targeting tumor acidity may have been applied to biologically heterogeneous patient populations in which only a subset of tumors were metabolically vulnerable.

Pharmacokinetic and delivery-related limitations also represent major translational barriers. Acidic and hypoxic tumor regions are frequently poorly perfused and exhibit elevated interstitial pressure, limiting drug penetration into the areas most strongly associated with extracellular acidosis [53,54,55,97]. This problem may be particularly important for agents targeting proton transport systems or metabolic pathways, where insufficient regional drug exposure can substantially reduce therapeutic efficacy despite systemic target engagement.

Clinical responses may also be limited by intratumoral heterogeneity and adaptive metabolic compensation. Even when acidity-related pathways are successfully inhibited, tumors may maintain viability through alternative metabolic programs or redundant pH-regulating mechanisms [4,25,42]. These adaptive responses are difficult to model fully in preclinical systems and may contribute to the relatively transient responses observed in clinical settings. Preclinical tumor models may also underestimate the degree of spatial heterogeneity, stromal complexity, and long-term metabolic adaptation observed in human cancers. Many experimental tumors are relatively homogeneous, rapidly growing, and metabolically less diverse than advanced human tumors, potentially exaggerating the apparent efficacy of acidity-targeting interventions in preclinical settings [3,25,82,94,96].

Importantly, several clinical studies have demonstrated measurable pharmacodynamic effects without corresponding survival benefit. For example, metabolic modulation with dichloroacetate and lactate transport inhibition with AZD3965 produced evidence of metabolic target engagement but limited and variable antitumor activity in early clinical studies [61,73,78]. These findings suggest that biochemical modulation of tumor acidity alone may not be sufficient to generate durable clinical responses.

### 4.7. Summary: A Reproducible Pattern of Translational Failure

Taken together, clinical studies of acidity-targeting strategies reveal a consistent and reproducible pattern. First, these interventions often achieve measurable biological effects, confirming target engagement. Second, clinical responses are typically modest, heterogeneous, and transient. Third, therapeutic efficacy is highly context-dependent, influenced by tumor type, microenvironmental heterogeneity, and compensatory mechanisms.

Importantly, these failures are not confined to a single class of therapeutic agents but are observed across multiple independent strategies. This convergence suggests that the limitations of targeting tumor acidity are not merely technical but may reflect deeper biological constraints.

These observations lead to a critical conclusion: the failure of acidity-targeting therapies is unlikely to be explained solely by suboptimal drug design. Instead, it points to a fundamental gap in our understanding of tumor acidity as a therapeutic target. In the following section, we explore this possibility by examining the underlying biological and translational barriers that limit the effectiveness of targeting tumor acidity. Clinical evaluations of these approaches are summarized in Table 3.

## 5. Why Targeting Tumor Acidity Fails: Mechanistic and Translational Barriers

### 5.1. Spatial Heterogeneity of Tumor pH

A fundamental limitation of targeting tumor acidity arises from the pronounced spatial heterogeneity of pH within tumors. Rather than being uniformly acidic, tumors exhibit steep pH gradients across microscopic and macroscopic scales, driven by variations in perfusion, oxygen availability, and metabolic activity [5,6,15,82,96].

Regions adjacent to blood vessels may maintain near-physiological pH, while poorly perfused or hypoxic regions can become highly acidic. As a result, therapies designed to target tumor acidity may only affect a subset of tumor cells, leaving other regions relatively unaffected. This spatial heterogeneity limits the overall therapeutic impact and contributes to incomplete tumor responses [53,55,94].

### 5.2. Temporal Dynamics and Adaptive Evolution

Tumor acidity is not a static feature but a dynamic property that evolves over time. Changes in tumor growth, vascular remodeling, and treatment-induced stress can alter metabolic activity and pH regulation [3,25,43,100].

Cancer cells can adapt to fluctuating pH conditions by reprogramming their metabolism and activating stress response pathways. For example, exposure to acidic environments can induce phenotypic changes that enhance invasiveness, resistance to apoptosis, and metabolic flexibility [24,42]. These adaptive responses reduce the long-term effectiveness of therapies targeting tumor acidity.

### 5.3. Metabolic Plasticity and Network Redundancy

One of the most significant barriers to targeting tumor acidity is the metabolic plasticity of cancer cells. While many tumors rely on aerobic glycolysis, they retain the ability to switch between metabolic pathways, including oxidative phosphorylation, glutaminolysis, and fatty acid oxidation [2,3,44,45,46,47,72].

This flexibility allows cancer cells to maintain energy production and redox balance even when glycolysis or lactate production is inhibited. In addition, multiple redundant pathways regulate intracellular and extracellular pH, including various proton transporters and buffering systems [8,13,29]. As a result, targeting a single metabolic or transport pathway is often insufficient to disrupt tumor acidity in a sustained manner.

### 5.4. Quantitative and Biological Constraints on Tumor Acidity

An additional limitation of current acidity-targeting strategies is that tumor acidity is often interpreted as a static biochemical feature rather than a dynamic systems-level variable. In reality, extracellular pH within tumors is continuously shaped by nonlinear interactions among metabolic flux, oxygen availability, vascular perfusion, proton transport, stromal buffering capacity, and immune-cell activity [4,5,8,15].

Experimental and computational studies suggest that tumor pH dynamics exhibit substantial spatial and temporal variability across tumor regions, generating heterogeneous microenvironmental states that may respond differently to therapy [4,82,92,94]. Importantly, relatively small alterations in perfusion or metabolic activity may produce disproportionately large local changes in extracellular acidity due to threshold-dependent and adaptive network behavior [4,29,100].

These observations indicate that tumor acidity cannot be fully understood through single-pathway models alone. Instead, systems-level frameworks integrating metabolic flux, diffusion dynamics, vascular architecture, and evolutionary adaptation may be required to predict therapeutic response more accurately [4,101,102,103]. Such approaches could also improve patient stratification by identifying tumors in which acidic microenvironmental states are both spatially dominant and therapeutically actionable [5,96,104].

From a translational perspective, incorporation of quantitative imaging, computational modeling, and longitudinal metabolic monitoring may help bridge the gap between biochemical target engagement and clinically meaningful therapeutic outcomes [5,25,86].

Several barriers discussed in this review, including metabolic plasticity and tumor heterogeneity, are broadly relevant to many metabolism-targeting therapies and are not unique to acidity-targeting approaches [3,44,72]. However, tumor acidity also presents several distinctive challenges. Unlike many metabolic targets, extracellular pH is strongly constrained by systemic physiological regulation and is spatially heterogeneous at both microscopic and regional scales [27,65,82]. In addition, the most acidic tumor regions are frequently poorly perfused and difficult to access therapeutically, creating a mismatch between target localization and effective drug delivery [53,54,55,97]. These features distinguish acidity-targeting strategies from many other forms of metabolic intervention.

### 5.5. Systemic Constraints and Host Physiology

Unlike many molecular targets, extracellular pH is tightly regulated at the level of the whole organism. Systemic acid–base homeostasis is maintained within a narrow physiological range through renal, respiratory, and buffering mechanisms [27,65].

This imposes a fundamental constraint on therapeutic strategies aimed at modulating tumor acidity. Interventions such as systemic buffering cannot selectively alter tumor pH without affecting normal tissues, limiting the achievable therapeutic window. Consequently, the degree of pH modulation that can be safely achieved in patients may be insufficient to produce meaningful antitumor effects [83,98].

### 5.6. Drug Delivery Barriers in Acidic and Hypoxic Regions

Paradoxically, the regions of tumors that are most acidic are often the least accessible to therapeutic agents. Poor vascularization, high interstitial pressure, and abnormal extracellular matrix architecture limit drug penetration into hypoxic and acidic tumor regions [53,54,55,97].

As a result, therapies targeting tumor acidity may fail to reach the very regions where they are most needed. This mismatch between drug distribution and target localization represents a critical barrier to effective treatment and is not adequately captured in many preclinical models [101,102,105].

### 5.7. Immunological Complexity and Context-Dependent Effects

Although tumor acidity is generally associated with immune suppression, its relationship with the immune system is complex and context-dependent. While lactate accumulation can inhibit effector T cell function, it may also influence other immune cell populations in ways that are not fully understood [19,22,41,77,106].

Moreover, reducing tumor acidity does not necessarily restore effective antitumor immunity. Immune responses are regulated by multiple factors, including antigen presentation, checkpoint signaling, and stromal interactions. As a result, targeting acidity alone may be insufficient to overcome the broader immunosuppressive network within the tumor microenvironment [76,95,107].

### 5.8. Conceptual Limitation: Acidity as a Consequence, Not a Driver

Perhaps the most fundamental limitation lies in how tumor acidity is conceptualized. While acidity contributes to multiple aspects of tumor progression, it is primarily a downstream consequence of underlying metabolic and microenvironmental processes rather than a sufficient standalone driver [2,3,108].

This distinction has important therapeutic implications. Targeting acidity alone may not address the upstream mechanisms that sustain tumor growth and survival. In this sense, tumor acidity may be better understood as a consequence of broader tumor microenvironmental adaptation rather than a discrete and isolatable therapeutic target [29,95]. This does not imply that tumor acidity lacks biological importance or therapeutic relevance. Rather, it suggests that acidity alone may be insufficient as a durable standalone therapeutic target because it reflects broader metabolic and microenvironmental states maintained by multiple interconnected processes [2,3,29,95].

Targeting downstream consequences of tumor metabolism is not necessarily ineffective. Indeed, several successful cancer therapies indirectly modulate downstream biological states rather than directly targeting initiating oncogenic events. However, downstream phenotypes such as extracellular acidosis may exhibit reduced therapeutic vulnerability when maintained by multiple compensatory pathways and adaptive microenvironmental interactions [4,29,42]. In this context, therapies directed against acidity alone may face intrinsic limitations unless combined with approaches targeting upstream metabolic regulation, immune suppression, or tumor vascular dynamics [76,95,107].

#### Why Preclinical Success Does Not Translate Clinically

One of the most consistent observations in acidity-targeting research is the discrepancy between strong preclinical efficacy and limited clinical benefit. Several factors may contribute to this gap. Many preclinical models do not fully reproduce the spatial and metabolic heterogeneity of human tumors, particularly with respect to hypoxia, vascular structure, immune composition, and nutrient gradients [53,54,55,82,92]. As a result, experimental tumors may appear more uniformly dependent on acidity-related pathways than clinical tumors [3,25,29].

In addition, human tumors possess substantial adaptive capacity. Under therapeutic pressure, cancer cells can switch between metabolic pathways and compensate through redundant pH-regulating mechanisms [4,42,44]. These adaptive responses may reduce the long-term impact of therapies targeting a single metabolic or proton transport pathway.

Some translational barriers also appear to be more clinically important than others. In particular, intratumoral heterogeneity, poor drug penetration into hypoxic regions, and the lack of reliable biomarkers for patient selection remain major limitations [53,54,55,92,96]. This may explain why several acidity-targeting strategies demonstrate measurable metabolic effects without producing durable clinical responses [13,25,86].

These limitations suggest that future therapeutic approaches will likely require integration of metabolic targeting with biomarker-guided stratification, immune modulation, and improved delivery strategies rather than direct pH modulation alone [76,95,101,107].

### 5.9. Summary: From Target to Emergent Property

Taken together, these factors suggest that the failure of acidity-targeting strategies is not due to a single limitation but arises from the interplay of multiple biological and physical constraints. Spatial heterogeneity, temporal dynamics, metabolic plasticity, systemic regulation, delivery barriers, and immunological complexity collectively limit the effectiveness of interventions aimed at modulating tumor acidity.

These observations support a shift in perspective: rather than viewing tumor acidity as a standalone therapeutic target, it should be understood as a consequence of a complex and adaptive system. Effective therapeutic strategies will therefore require approaches that account for this complexity, rather than attempting to neutralize acidity in isolation. These translational barriers are summarized in Table 4.

### 5.10. Rethinking Tumor Acidity as a Consequence of Tumor Adaptation

The limited clinical success of acidity-targeting therapies may reflect not only technical limitations, but also the way tumor acidity has been conceptualized therapeutically. Unlike oncogenic driver mutations or lineage-dependent signaling pathways, extracellular acidity does not arise from a single dominant molecular event. Instead, it emerges through interconnected processes involving metabolic activity, hypoxia, vascular dysfunction, stromal interactions, and adaptive stress responses within the tumor microenvironment. Consequently, therapies directed against a single acidity-generating mechanism may have limited effectiveness because extracellular acidosis is continuously sustained through compensatory and system-level adaptive processes [2,3,13,25,42,95]. These observations suggest that tumor acidity may be better understood as a consequence of broader metabolic and microenvironmental adaptation rather than as an isolated therapeutic target [2,3,29,95,108].

Extracellular acidification does not result from a single dominant pathway. Instead, it develops through the combined effects of metabolic reprogramming, hypoxia, vascular dysfunction, stromal metabolic interactions, immune suppression, and adaptive responses within the tumor microenvironment [3,16,17,19,29,41]. These processes are closely linked and can compensate for one another under therapeutic pressure [4,25,42].

This perspective helps explain why therapies that successfully modulate lactate levels, proton transport, or extracellular pH frequently fail to produce durable clinical responses [13,25,86]. Although such interventions may alter local biochemical parameters, they often do not sufficiently disrupt the upstream network architecture that continuously regenerates and stabilizes the acidic microenvironment [29,95,102].

Viewing tumor acidity as a consequence of broader tumor adaptation has important therapeutic implications. Rather than attempting to directly eliminate extracellular acidity, future strategies may need to combine metabolic targeting with approaches affecting tumor vasculature, stromal interactions, and antitumor immunity [76,95,101,107,109]. From this perspective, tumor acidity may be more useful as an indicator of the metabolic and microenvironmental state of tumors than as an isolated therapeutic target [29,103,108].

## 6. What Works: Emerging Strategies to Overcome Translational Barriers

### 6.1. Combination Therapies Targeting Metabolism and Immunity

Given the limited efficacy of monotherapies targeting tumor acidity, combination strategies have emerged as a more promising approach. In particular, integrating metabolic interventions with immunotherapy has shown considerable potential. Preclinical studies demonstrate that reducing lactate accumulation can restore T cell function, enhance cytokine production, and improve responses to immune checkpoint blockade [19,21,22,38,110].

These findings suggest that tumor acidity acts as a metabolic checkpoint that suppresses antitumor immunity. By combining acidity-targeting strategies with immune checkpoint inhibitors, it may be possible to overcome this suppression and achieve more durable therapeutic responses [50,76,77,111]. Early clinical investigations exploring such combinations are ongoing, although optimal dosing, timing, and patient selection remain to be defined.

### 6.2. pH-Responsive Drug Delivery Systems

Rather than attempting to neutralize tumor acidity, an alternative strategy is to exploit it. pH-responsive drug delivery systems are designed to selectively release therapeutic agents in acidic environments, thereby enhancing tumor specificity while minimizing systemic toxicity [28,69,71,112].

These systems include pH-sensitive nanoparticles, polymeric carriers, and prodrugs that undergo structural or chemical changes in response to low pH. Preclinical studies have demonstrated improved drug accumulation and enhanced antitumor efficacy using such approaches [71,74,75,113]. Importantly, this strategy leverages tumor acidity as a targeting mechanism rather than attempting to eliminate it, potentially bypassing some of the limitations associated with systemic pH modulation [74,99,114].

Several emerging studies suggest that acidity-targeting approaches may be more effective when integrated with other therapeutic modalities rather than used as standalone interventions. For example, inhibition of lactate transport or proton regulation has shown improved efficacy when combined with immune checkpoint blockade or radiotherapy in preclinical models [22,61,76]. Similarly, pH-responsive delivery systems and acid-activated prodrugs may allow selective drug release within acidic tumor regions while minimizing systemic toxicity [28,68,69,70]. These approaches do not attempt to eliminate tumor acidity directly, but instead exploit acidic microenvironments as a therapeutic vulnerability.

### 6.3. Microenvironment Reprogramming Strategies

Another emerging approach involves reprogramming the tumor microenvironment rather than directly targeting acidity. This includes strategies aimed at modulating stromal cells, such as cancer-associated fibroblasts (CAFs), as well as altering immune cell function and metabolic interactions within the TME [17,19,56,109].

By disrupting the metabolic crosstalk that contributes to acid production—such as lactate exchange between tumor and stromal cells—it may be possible to indirectly reduce tumor acidity and its associated pro-tumorigenic effects [18,41,106,115]. These approaches reflect a shift from targeting individual components to modifying the broader system that generates and maintains the acidic microenvironment.

### 6.4. Biomarker-Guided Patient Stratification

One of the key challenges in translating acidity-targeting strategies is the lack of reliable biomarkers for patient selection. Tumor acidity is highly heterogeneous, and not all tumors exhibit the same degree of dependence on glycolysis or lactate metabolism [3,25,75].

Emerging approaches aim to address this limitation by incorporating metabolic and imaging-based biomarkers to identify patients who are most likely to benefit from acidity-targeting therapies. Techniques such as magnetic resonance-based pH imaging and metabolic profiling offer potential tools for assessing tumor acidity in vivo [5,115].

Importantly, integrating metabolomic signatures into clinical decision-making may enable a more precise and personalized approach to targeting tumor acidity, aligning therapeutic strategies with the metabolic phenotype of individual tumors [45,47,96].

### 6.5. Emerging Technologies for Characterizing Acidic Tumor Microenvironments

Advances in spatial and computational approaches may help address some of the limitations associated with acidity-targeting therapies. In particular, techniques such as spatial omics, computational modeling, and mechanobiological analysis provide new ways to examine metabolic heterogeneity and microenvironmental organization within tumors [92,93,94,96,101].

Spatial transcriptomic and metabolomic approaches have revealed substantial intratumoral heterogeneity in metabolic states, immune-cell distribution, hypoxia, and stromal organization, demonstrating that tumor acidity is neither spatially uniform nor biologically static [92,93,94,96]. These technologies may enable identification of localized acidic niches that are more therapeutically vulnerable and may improve patient stratification for metabolism-targeted therapies.

Artificial intelligence (AI) and machine learning approaches are also increasingly being applied to integrate multimodal datasets, including metabolic imaging, spatial profiling, and clinical outcome data [101,103]. Such integrated computational models may improve prediction of tumor regions that are likely to exhibit therapy-resistant acidic microenvironments and may help optimize adaptive or combination treatment strategies. AI-assisted integration of spatial metabolomics, pH imaging, transcriptomics, and immune-cell topology may enable identification of tumor regions in which extracellular acidity is functionally linked to immune suppression, metabolic vulnerability, or therapeutic resistance. Such multidimensional approaches could improve biomarker-guided patient stratification and support adaptive treatment strategies based on dynamic microenvironmental states rather than static molecular features alone [5,92,96,101].

In parallel, mechanobiological studies suggest that extracellular matrix stiffness, interstitial pressure, and vascular compression contribute substantially to impaired perfusion and metabolite diffusion within tumors [53,54,55,97]. These physical constraints may reinforce local acid accumulation and limit drug delivery into hypoxic regions. Integrating mechanobiological parameters with metabolic and spatial analyses may therefore provide a more comprehensive framework for understanding acidity-associated therapeutic resistance.

Together, these approaches may improve understanding of how acidic microenvironments develop and change within tumors and may help guide more precise therapeutic strategies.

### 6.6. Targeting System-Level Properties Rather than Single Pathways

Clinical experience suggests that tumor acidity is unlikely to be effectively controlled through single-pathway interventions alone. More effective approaches may require combined targeting of multiple components of the tumor microenvironment, including metabolism, vascular function, and immune regulation [13,29,116].

Approaches incorporating multi-target therapies, adaptive treatment strategies, and broader microenvironmental modulation may help address the complexity and redundancy of tumor biology [101,102,103,109,117]. These strategies are based on the idea that tumor acidity develops through multiple interacting biological processes and is therefore difficult to control through inhibition of a single pathway alone.

Future therapeutic strategies will likely require integration of metabolic targeting with biomarker-guided patient selection, imaging-based monitoring, and immune modulation rather than acidity-targeting monotherapy alone [22,76,77,96]. In particular, longitudinal assessment of tumor metabolic states and spatial heterogeneity may help guide adaptive treatment strategies and improve identification of tumors that are more dependent on acidity-associated pathways [5,92,115]. These approaches may ultimately support more precise and context-dependent therapeutic intervention within metabolically heterogeneous tumor microenvironments.

### 6.7. Summary: From Elimination to Exploitation

Recent advances suggest that future therapeutic approaches may need to move beyond direct acidity neutralization alone. Rather than attempting to eliminate tumor acidity, which is constrained by biological and systemic limitations, more effective approaches may involve exploiting acidity as a functional feature of the tumor microenvironment or integrating it into multi-modal treatment strategies.

This shift reflects a broader transition in cancer therapy—from targeting isolated molecular pathways to understanding and manipulating complex biological systems [95,102,107].

### 6.8. Hypothesis-Driven Future Directions

The limited clinical success of acidity-targeting therapies suggests that future studies should move beyond direct pH neutralization alone. One possibility is that therapeutic response depends less on the absolute level of extracellular acidity and more on how effectively tumor cells adapt to acidic stress [24,25,42]. Tumors with reduced metabolic flexibility may therefore be more sensitive to therapies targeting acid-associated metabolic adaptation.

A second emerging hypothesis is that acidity-associated immune dysfunction may be reversible only when metabolic interventions are combined with restoration of immune-cell bioenergetic fitness [21,22,40,76]. This possibility suggests that successful immunometabolic therapies may require simultaneous targeting of tumor lactate metabolism and T-cell metabolic resilience rather than extracellular acidity alone.

Another critical area for future investigation involves the spatial organization of acidic tumor niches. Increasing evidence suggests that localized acidic microenvironments may function as evolutionary reservoirs for therapy-resistant and immune-evasive tumor cell populations [4,92,94]. Future studies integrating spatial metabolomics, longitudinal imaging, and computational modeling may therefore help identify tumor regions in which acidity contributes disproportionately to malignant progression and treatment resistance [5,96,101].

Finally, future clinical development may benefit from reframing tumor acidity not primarily as a direct therapeutic target, but as a systems-level biomarker reflecting metabolic adaptation, vascular dysfunction, and microenvironmental stress [29,95,108]. This perspective may support more precise patient stratification and improve the rational design of combination therapies targeting broader microenvironmental and metabolic interactions rather than isolated metabolic pathways.

## 7. Conclusions

Tumor acidity has long been recognized as a hallmark of cancer and a promising therapeutic target. Extensive preclinical research has demonstrated that modulating tumor pH or lactate metabolism can inhibit tumor growth, suppress metastasis, and enhance immune responses. However, these promising findings have not translated into consistent clinical success.

In this review, we have examined this translational paradox and identified key factors that contribute to the failure of acidity-targeting strategies. These include spatial and temporal heterogeneity, metabolic plasticity, systemic physiological constraints, drug delivery barriers, and the complex interplay between tumor cells and the immune microenvironment. Together, these findings suggest that tumor acidity is closely linked to broader metabolic and microenvironmental changes within tumors rather than functioning as an isolated therapeutic target.

This perspective has important implications for future therapeutic development. Strategies focusing only on single pathways or direct pH neutralization may have limited long-term clinical benefit. Instead, successful approaches will require integration across multiple dimensions of tumor biology, including metabolism, immunity, and microenvironmental structure.

Ultimately, the limited clinical success of acidity-targeting therapies may reflect not only technical limitations in drug development, but also a conceptual limitation in how tumor acidity has been therapeutically framed. Improved understanding of how tumor acidity develops within different tumor microenvironments may help guide future combination and biomarker-based therapeutic strategies.

## Figures and Tables

**Table 1:** Mechanisms contributing to extracellular acidosis in tumors. Summary of the major biological mechanisms that generate and maintain extracellular acidosis in the tumor microenvironment. The table integrates metabolic pathways, proton transport systems, hypoxia-driven processes, and stromal interactions, linking each mechanism to its biological consequences and therapeutic implications.

Mechanism	Key Molecular Players	Contribution to Extracellular Acidosis	Biological Consequence	Therapeutic Implication	Major Limitation	Key Reference
**Aerobic glycolysis (Warburg effect)**	LDHA, PKM2, HIF-1α	Increased lactate and proton production	Acidic microenvironment, metabolic reprogramming	Target glycolysis or lactate production	Metabolic plasticity and compensation	[2,3,7,10]
**Lactate export**	MCT1, MCT4	Extrusion of lactate and H^+^	Establishment of acidic extracellular pH	Target lactate transport	Transporter redundancy (MCT4 compensation)	[12,18,26,50]
**Hypoxia-driven metabolism**	HIF-1α, VEGF	Increased glycolysis and reduced clearance	Acid accumulation and immune suppression	Target hypoxia pathways	Spatial heterogeneity of hypoxia	[15,16,48]
**Proton transport systems**	NHE1, V-ATPase	Active proton extrusion	Maintenance of reversed pH gradient	Target proton pumps and exchangers	Redundant pH regulation systems	[8,13,51]
**Carbonic anhydrase activity**	CAIX, CAXII	Conversion of CO_2_ to H^+^ and HCO_3_^−^	Stabilization of acidic microenvironment	Target CAIX in hypoxic tumors	Limited specificity and delivery issues	[31,32,52]
**Poor perfusion and clearance**	Abnormal vasculature	Accumulation of acidic metabolites	Local acidosis and therapy resistance	Normalize vasculature	Drug delivery limitations	[53,54,55]
**Stromal metabolic crosstalk**	CAFs, immune cells	Lactate exchange and metabolic coupling	Reinforced acidity and tumor progression	Target stromal interactions	System-level complexity	[17,19,41,56]

**Table 2:** Preclinical strategies targeting tumor acidity and their limitations. Overview of representative preclinical approaches designed to target tumor acidity. The table highlights experimental models, observed antitumor effects, proposed mechanisms of action, and key limitations that have hindered successful clinical translation.

Strategy Category	Representative Target/Approach	Experimental Model	Main Preclinical Outcome	Proposed Mechanism	Major Limitation (Translational)	Key Reference
**Metabolic inhibition**	LDHA inhibition	Cell lines, xenografts	Reduced tumor growth, lactate production	Block glycolysis	Metabolic plasticity	[29,57,58,72]
**Metabolic modulation**	DCA	Mouse models	Shift to OXPHOS, reduced lactate	PDH activation	Limited tumor dependency	[59,73]
**Lactate transport inhibition**	MCT1 inhibition	Xenografts	Disrupted metabolic symbiosis	Block lactate exchange	Transporter redundancy	[18,50,60,61]
**Buffer therapy**	Sodium bicarbonate	Mouse models	Increased tumor pH, reduced metastasis	Neutralize extracellular acidity	Systemic constraints	[27,64,65]
**Proton transport inhibition**	V-ATPase, NHE1 inhibitors	In vitro/in vivo	Intracellular acidification, tumor suppression	Disrupt pH homeostasis	Redundant pathways	[13,51,66]
**CAIX inhibition**	CAIX inhibitors	Xenografts	Reduced tumor growth in hypoxia	Block pH regulation	Delivery limitations	[31,52,67]
**pH-responsive delivery**	Nanoparticles, prodrugs	Animal models	Enhanced drug accumulation	Acid-triggered release	Translation complexity	[68,70,71,74,75]
**Immunometabolic targeting**	Lactate inhibition + immunotherapy	Mouse models	Improved T cell function	Reverse immune suppression	Limited clinical translation	[19,21,22,76,77]

**Table 3:** Clinical evaluation of acidity-targeting strategies and outcomes. Summary of major therapeutic strategies evaluated in clinical or translational settings. The table outlines representative agents, target pathways, clinical contexts, and key findings, with emphasis on the limited and inconsistent efficacy observed across different approaches.

Strategy Class	Agent/Intervention	Target/Pathway	Cancer Type	Trial Phase/Study Type	Combination or Monotherapy	Key Findings	Major Limitation/Reason for Limited Efficacy	Clinical Status	Key Reference
**Metabolic modulation**	Dichloroacetate (DCA)	PDH activation (glycolysis → OXPHOS shift)	Glioblastoma, solid tumors	Early clinical/pilot studies	Monotherapy	Metabolic shift observed; limited tumor regression	Metabolic plasticity; insufficient tumor dependency on glycolysis	Limited clinical activity	[3,73]
	LDHA-targeting strategies (various early agents)	Lactate production inhibition	Solid tumors	Preclinical → early clinical exploration	Mostly monotherapy	Reduced lactate (preclinical); limited clinical data	Redundant metabolic pathways; compensation via OXPHOS/glutamine	Early-stage/no clear efficacy	[2,57,72]
**Lactate transport inhibition**	AZD3965	MCT1 inhibitor	Lymphoma, solid tumors	Phase I	Monotherapy ± combination	Target engagement confirmed; modest responses	MCT4 compensation; tumor heterogeneity	Early clinical/ongoing	[61,63,78]
	MCT inhibitors (class)	Lactate export blockade	Various solid tumors	Early clinical exploration	Monotherapy	Metabolic disruption observed	Transporter redundancy; context dependency	Limited efficacy	[26,50,82]
**pH regulation machinery**	CAIX inhibitors (e.g., SLC-0111)	Carbonic anhydrase IX	Solid tumors (hypoxic tumors)	Phase I/II	Monotherapy/combination	Good tolerability; limited efficacy signals	Tumor heterogeneity; limited drug penetration	Early clinical/ongoing	[31,52,85]
	Proton pump inhibitors (e.g., omeprazole)	V-ATPase inhibition	Various solid tumors	Small clinical studies	Combination (chemo)	Some sensitization reported	Lack of specificity; systemic effects	Inconclusive	[51,83,98]
	NHE1 inhibitors (experimental)	Na^+^/H^+^ exchange	Preclinical → limited clinical	Early-stage	Monotherapy	Intracellular acidification (preclinical)	Redundancy of pH regulation systems	Not clinically established	[13,42]
**Systemic buffering**	Sodium bicarbonate	Extracellular pH neutralization	Solid tumors (preclinical → limited clinical attempts)	Translational/exploratory	Monotherapy	Increased tumor pH (preclinical)	Systemic pH constraint; poor tumor selectivity	Not clinically viable	[27,64,65]
**Hypoxia-linked approaches**	Hypoxia-activated prodrugs (e.g., tirapazamine)	Hypoxia-associated acidity	Head and neck, lung cancer	Phase II/III	Combination (RT/chemo)	Mixed clinical outcomes	Hypoxia heterogeneity; patient selection issues	No consistent benefit	[87,88,91]
	HIF pathway inhibitors	Hypoxia-driven metabolism	Various solid tumors	Early clinical	Monotherapy/combination	Biological activity observed	Complex network regulation; indirect effect on acidity	Limited efficacy	[16,89]
**Acidity-exploiting strategies**	pH-responsive drug delivery systems	Acid-triggered drug release	Various solid tumors (early trials)	Early clinical/translational	Combination	Improved tumor targeting (early evidence)	Delivery complexity; scalability	Emerging	[28,70,74,99]
**Immunometabolic combination**	Lactate targeting + checkpoint inhibitor	Metabolism + immune suppression	Various solid tumors	Preclinical → early clinical	Combination	Enhanced immune response (preclinical)	Limited clinical validation; complexity	Early-stage	[22,76,77]

**Table 4:** Translational barriers limiting the clinical efficacy of acidity-targeting therapies. Overview of the major biological and physiological barriers that constrain the clinical success of therapies targeting tumor acidity. The table connects underlying mechanisms to their clinical manifestations and implications for future trial design, emphasizing the multifactorial nature of translational failure.

Translational Barrier	Biological Basis	Clinical Manifestation	Affected Strategy Classes	Implication for Future Trial Design	Key Reference
**Intratumoral spatial heterogeneity**	Uneven perfusion, oxygen gradients, and regional metabolic variation generate heterogeneous pH distribution	Partial or inconsistent tumor responses; resistant regions persist	Hypoxia-targeted therapies, MCT inhibitors, CAIX inhibitors	Incorporate imaging-guided targeting and region-specific delivery strategies	[6,15,82,96]
**Temporal dynamics and tumor evolution**	Tumor metabolism and pH regulation adapt over time under therapeutic pressure	Initial response followed by rapid resistance or relapse	Metabolic modulators (DCA, LDHA inhibitors)	Design adaptive or sequential treatment strategies; longitudinal monitoring required	[3,16,25]
**Metabolic plasticity**	Flexible switching between glycolysis, oxidative phosphorylation, and alternative substrates	Maintenance of tumor viability despite metabolic inhibition	LDHA inhibitors, DCA, glycolysis-targeting approaches	Combine multi-pathway metabolic interventions rather than single-target strategies	[2,3,44,45,72]
**Redundancy of pH regulation systems**	Multiple overlapping proton transport systems (MCTs, NHE1, CAIX, V-ATPases) maintain pH homeostasis	Limited efficacy of single-target inhibitors due to compensatory mechanisms	MCT inhibitors, CAIX inhibitors, proton pump inhibitors	Develop multi-target or network-level inhibition strategies	[8,13,29,42]
**Systemic physiological constraints**	Tight regulation of systemic acid–base balance by renal and respiratory systems	Inability to achieve sufficient tumor-specific pH modulation	Buffer therapy (e.g., bicarbonate)	Focus on tumor-selective targeting or localized delivery approaches	[27,65,83]
**Drug delivery limitations**	Poor vascularization, high interstitial pressure, and dense extracellular matrix restrict drug penetration	Insufficient drug exposure in hypoxic and acidic tumor regions	CAIX inhibitors, hypoxia-targeted drugs, metabolic inhibitors	Improve delivery systems (nanoparticles, targeted carriers) to reach poorly perfused regions	[53,54,55,97,102]
**Pharmacodynamic–clinical disconnect**	Biochemical modulation of pH or lactate does not directly translate to tumor regression	Changes in metabolic biomarkers without survival benefit	AZD3965, DCA, metabolic targeting agents	Redefine clinical endpoints beyond metabolic readouts; integrate functional outcomes	[13,25,86]
**Immunological complexity**	Tumor immunity is regulated by multiple interacting pathways beyond acidity alone	Limited restoration of antitumor immunity despite lactate reduction	Lactate-targeting strategies, metabolic inhibitors	Combine with immunotherapy (e.g., checkpoint inhibitors)	[19,22,36,38]
**Tumor-type dependency**	Variable reliance on glycolysis and lactate metabolism across tumor types	Context-dependent responses across different cancers	MCT inhibitors, metabolic therapies	Implement biomarker-guided patient selection strategies	[3,82,96]
**Conceptual limitation (acidity as a consequence)**	Tumor acidity reflects downstream effects of broader metabolic and microenvironmental processes	Targeting acidity alone fails to disrupt upstream drivers of tumor progression	Nearly all acidity-targeting approaches	Shift focus toward system-level interventions and upstream pathway modulation	[2,3,95,108]

## Data Availability

No new data were created or analyzed in this study. Data sharing is not applicable to this article.

## Abbreviations

CA	Carbonic anhydrase
CAFs	Cancer-associated fibroblasts
CAIX	Carbonic anhydrase IX
CTLs	Cytotoxic T lymphocytes
DCA	Dichloroacetate
HIF-1α	Hypoxia-inducible factor 1-alpha
LDHA	Lactate dehydrogenase A
MCT	Monocarboxylate transporter
MDSCs	Myeloid-derived suppressor cells
NHE1	Na^+^/H^+^ exchanger 1
NK cells	Natural killer cells
OXPHOS	Oxidative phosphorylation
PDH	Pyruvate dehydrogenase
pHe	Extracellular pH
pHi	Intracellular pH
TME	Tumor microenvironment
V-ATPase	Vacuolar H^+^-ATPase
